# Supplementary material for: Gamma distribution model of diffusion MRI for the differentiation of primary central nerve system lymphomas and glioblastomas
Source: PLoS One. 2020 Dec 14;15(12):e0243839. doi: 10.1371/journal.pone.0243839 (PMC7737570; doi:10.1371/journal.pone.0243839)
Supplement: Attachment — Submitted filename: Response to Reviewers.docx [file pone.0243839.s002.docx]

First of all, we would like to thank the two reviewers for their thorough reading and constructive criticism which greatly enhanced the quality of the paper. According to the comments, we have carefully revised the paper.

Reviewer #1:----Summary
The manuscript reports the use of the diffusion MRI gamma distribution (GD) model in the differentiation of primary central nervous system lymphomas (PCNSLs) and glioblastomas (GBs).
The results indicate that the GD model is advantageous in terms of diagnostic performance when comparing these 2 lesions, namely when 3 specific parameters of this model are used in combination.
My overall impression is that the manuscript has quality and it is an interesting an innovative topic, but some aspects should be considered and better analysed.

----Comments to the Author

• Abstract:
Page 2 - “In this study, we investigate whether whether the gamma distribution (GD) model is useful in this differentiation of PNCSLs. and GBs” – The word “whether” is repeated and the full stop after PNCSLs should be removed.

**Response:** Thank you for pointing out. These have been eliminated.

Page 2 - “Receiver operating curve (ROC) analyses were performed to assess diagnostic performance.” – ROC stands for Receiver Operating Characteristic. This should appear as: Receiver Operating Characteristic (ROC) curve. Please check the rest of the document, as this is recurrent.

**Response:** Thank you very much. This has been corrected.

• Introduction:
Page 4 – Last paragraph – “The bi-exponential intravoxel incoherent motion (IVIM) model aims to separate the true water diffusion and the capillary perfusion by using multiple b-values [10, 11].” – multiple b-values but specifically multiple low b-values. Please add the word “low”

**Response:** We agree with this point. The term “low” has been added here.

There are only a few articles in this topic, and the way the authors composed parts of the introduction is very similar to the reference number 22 (Shinmoto et al. 2014) and/or 24 (Borlinhas et al. 2019 ) of the document, and sometimes being exactly the same, but not being cited.

Example 1
“The IVIM model has an associated uncertainty to the estimated pseudodiffusion, and perfusion fraction, and a possible overparametrization of the model.7 The limitations of the DKI model are related to the unclear biological interpretation of the mean kurtosis parameter, and to the effects of the high b-values that the model requires.” – Reference 24 of the manuscript
“The bi-exponential model could be influenced by an uncertainty of the estimated perfusion, since signal measurements at low b-values are susceptible to measurement errors [15-17]. The DKI model is limited by the unclear biological interpretation of the kurtosis parameters [18-20].” – The actual text in the manuscript

**Response:** The reference #18 (previous #24, Borlinhas et al.) has been added and cited for these sentences as suggested.

Example 2
“The GD model allows us to estimate fractions of a tissue type based on the concept that the area fractions for D <1.0 × 10−3 mm2/sec, 1.0 × 10−3 to 3.0 ×10−3 mm2/sec, and D >3.0 ×10−3 mm2/sec are attributed to intracellular, extracellular extravascular, and intravascular spaces, respectively [21].” – The actual text in the manuscript

– “ADC values smaller than 1.0 mm2/scan be attributed to small tumor cells with restricted diffusion, and ADC values larger than 3.0 mm2/s can be attributed to perfusion, with ADC values between 1.0 mm2/sand 3.0 mm2/s attributed to water diffusion in the other components.” – Reference 21 of the manuscript
– “(…)extracellular fluid (ADCs between 1.0 and 3.0 mm2/s)" – Reference 22 of the manuscript
– “Through the PDF of ADC, three different areas under the function’s curve are defined as follows: the fraction of diffusion lower than 1.00 × 10-3 mm2/s is the f1 fraction and it reflects the small cell component; the fraction of diffusion higher than 3.00 × 10-3 mm2/s is the f3 fraction and it reflects the perfusion component; and the fraction of diffusion between 1.00 × 10-3 mm2/s and 3.00 × 10-3 mm2/s is the f2 fraction translating the extracellular component of the tissue.12,13” - Reference 24 of the manuscript.
In this case, and as you can see, reference 21 is not specific when mentioning the meaning of f2. You have more specific references such as number 22 and 24 of your document.
Please add these references.

**Response:** We have added the two references (Borlinhas F, et al. and Shinmoto H, et al.) for the sentence “The GD model allows us to estimate fractions of a tissue type based on the concept that the area fractions for D <1.0 × 10^−3^ mm_2_/sec, 1.0 × 10^−3^ to 3.0 ×10^−3^ mm_2_/sec, and D >3.0 ×10^−3^ mm_2_/sec are attributed to intracellular, extracellular extravascular, and intravascular spaces, respectively [18, 22, 23]”

Page 5 - “The GD model allows us to estimate fractions of a tissue type based on the concept that the area fractions for D <1.0 × 10−3 mm2/sec, 1.0 × 10−3 to 3.0 ×10−3 mm2/sec, and D >3.0 ×10−3 mm2/sec are attributed to intracellular, extracellular extravascular, and intravascular spaces, respectively [21]” – This part “1.0 × 10−3 to 3.0 ×10−3 mm2/sec” must appear as “D=1.0 × 10−3 to 3.0 ×10−3 mm2/sec”

**Response:** This has been corrected to “D =1.0 × 10^−3^ to 3.0 ×10^−3^ mm^2^/sec” as suggested.

Page 5 – “The GD model is suitable for realistically interpreting diffusion data in a histological context.” - This sentence is very abrupt. If the reference you are using is the reference 21, Oshio et al. cautiously concluded the following: “(…) histological interpretation of the data appears possible.” In Oshio et al. work, the peripheral zone of the prostate and a prostate cancer were being compared, not different histological types of lesions for example. Your sentence can be misinterpreted.
Also, this sentence that you present should be connect to the information included in this paragraph.

**Response:** We agree with this point. We want to state that these fractions (f1, f2, f3) allow us to estimate histological conditions of tumors and organs. For example, tumors with high f1, f2, and f3 should have high cell density, large interstitial space, and high vascularity, respectively. So, this sentence has been replaced by the following one. We think this sentence is now natural in the context.

Introduction (Page 5, line 17-18)

“Based on these fractions, we may be able to estimate histopathological conditions of neoplasms or organs.”

Page 5 – “The GD model has been used to assess prostate cancers [21-23], breast cancers [24], and renal function [25]. To the best of our knowledge, its application to brain tumours has never been reported. We conducted the present study to determine whether the GD model is useful in the differentiation of PCNSLs and GBs.” – To the best of my knowledge that is true, but the GD model has been applied to brain studies. This is a relatively new application, and consequently, it would be worth mentioning this fact. Here is the a reference that you can use: Grinberg F, Farrher E, Ciobanu L, Geffroy F, Le Bihan D, et al. (2014) Non-Gaussian Diffusion Imaging for Enhanced Contrast of Brain Tissue Affected by Ischemic Stroke. PLoS ONE 9(2): e89225. doi:10.1371/journal.pone.0089225

**Response:** Thank you very much for the reference. We have modified the paragraph as follows. We would like to keep the sentence “To the best of our knowledge...” since the application to brain “tumors” have never been reported yet.

Introduction (Page 5, Line 20 – Page 6, Line 2)

“The GD model has been used to assess prostate cancers [22-24], breast cancers [18], and renal function [25]. The GD model was also used to assess cerebral ischemic stroke in rat brains, and it was showed that this model exhibited a better performance than the conventional mono-exponential model and allowed for a significantly enhanced visualization of ischemic lesions [26]. To the best of our knowledge, its application to brain tumors has never been reported. We conducted the present study to determine whether the GD model is useful in the differentiation of PCNSLs and GBs.”

New reference

26.	Grinberg F, Farrher E, Ciobanu L, Geffroy F, Le Bihan D, Shah NJ. Non-Gaussian diffusion imaging for enhanced contrast of brain tissue affected by ischemic stroke. PLoS One. 2014;9(2):e89225. Epub 2014/03/04. doi: 10.1371/journal.pone.0089225. PubMed PMID: 24586610; PubMed Central PMCID: PMCPMC3937347.

• Material and Methods
- Patients
Page 6 - “Multi-b-value DWI” – It would be more adequate to read for example “The use of a DWI protocol with multiple b-values (…)”

**Response:** Thank you for your suggestion. We have changed the wording as follows.

Materials and Methods (Page 6, Line 9, 11)

“The DWI protocol with multiple b-values has been a part of our routine preoperative MRI examination for patients with brain tumors since January 2013.”

“The DWI with multiple b-values was conducted preoperatively for the patient during the period from January 2013 to August 2019.”

Page 6 – The difference between the number of PCNSLs and GBs is relevant. Knowing that the PCNSLs are less frequent when compared to the GBs, this information could be added to justify the difference in the groups.

**Response:** Yes, the MLs are less frequent compared to GBs. The following sentence and a new reference have been added.

Materials and Methods (Page 6, Line 20-21)

“The difference between the number of patients with PCNSLs and GBs can be explained by the fact that the PCNSLs are less frequent compared to the GBs [27].”

New reference

27.	Ostrom QT, Cioffi G, Gittleman H, Patil N, Waite K, Kruchko C, et al. CBTRUS Statistical Report: Primary Brain and Other Central Nervous System Tumors Diagnosed in the United States in 2012-2016. Neuro Oncol. 2019;21(Suppl 5):v1-v100. Epub 2019/11/02. doi: 10.1093/neuonc/noz150. PubMed PMID: 31675094; PubMed Central PMCID: PMCPMC6823730.

If available, the characteristics of the GB, like fraction of necrosis or haemorrhage should be reported. The addition of these characteristics would be enriching the study and could explain some outliers in the results.

**Response:** Thank you very much for your kind suggestion. But, in the present study, we excluded necrosis or hemorrhage in the ROI-based measurements since we would like to evaluate tumorous lesions. It is possible to report the fraction of necrosis or frequency of hemorrhage, but I am afraid it may be confusing for readers since we avoided the areas with necrosis or hemorrhage in the measurements.

- ROI placement
Page 9 – The legend of this figure 1 repeats the information that it is presented in the text: “The ROIs were also placed on the noncontrast-enhancing T2-hyperintense areas as well as on the contralateral normal-appearing white matter on the image obtained with the b-value of 0 sec/mm2 image.”
The reason why and the purpose of placing ROIs on the noncontrast-enhancing T2-hyperintense areas should be stated.

**Response:** The legend of Figure 1 has been summarized to avoid duplications with the text. The reason why we examined peritumoral noncontrast-enhancing T2-hyperintense areas was to evaluate if there were histological differences such as tumor infiltration or increased vascularity in peritumoral regions between PCNSLs and GBs. There have been several studies that showed increased rCBV on DSC-perfusion imaging in peritumoral noncontrast-enhancing T2-hyperintense areas of GBs. This means that such areas include not only vasogenic edema but also tumor cells infiltrating surrounding brain parenchyma; however, our study did not reveal any significant differences in GD model-based parameters for peritumoral noncontrast-enhancing T2-hyperintense areas between PCNSLs and GBs. We assume that the high f2 values observed in peritumoral noncontrast-enhancing T2-hyperintense areas compared to enhancing areas in both types of tumor are likely to reflect perifocal vasogenic edema rather than tumor infiltration outside the enhancing lesion. We have modified the sentence in Materials and Methods and Discussion as follows.

**Fig. 1.** Region-of-interest (ROI). The ROIs were placed on postcontrast T1-weighted images to include contrast enhancing lesions (A, C). The ROIs were also placed on the non-contrast-enhancing T2-hyperintense areas surrounding the contrast-enhancing area and the contralateral normal-appearing white matter (B, D).

Materials and Methods (Page 9, Line 14-21)

“The ROIs were also placed on the peritumoral non-contrast-enhancing T2-hyperintense areas to evaluate whether there were differences in histological features including tumor infiltration or increased vascularity in the peritumoral areas between PCNSLs and GBs. In addition, the ROIs were placed on the contralateral normal-appearing white matter. The ROIs for the peritumoral non-contrast-enhancing T2-hyperintense areas and contralateral normal-appearing white matter were measured on the image obtained with the b-value of 0 sec/mm^2^ image. The same ROIs were used for all DWI analyses.”

Discussion (Page 23, Line 11- Page 24, Line 3)

“In the T2-hyperintense lesions without contrast enhancement, no significant differences were observed between the PCNSL and GB groups for any parameters. There have been several studies that showed increased rCBV on DSC-perfusion imaging in peritumoral noncontrast-enhancing T2-hyperintense areas of GBs [36, 37]. The results of these studies indicated that the peritumoral areas of GB include not only vasogenic edema but also tumor cells infiltrating surrounding brain parenchyma; however, our study did not reveal any significant differences in the GD model-based parameters for peritumoral noncontrast-enhancing T2-hyperintense areas between PCNSLs and GBs. The f2 values in the noncontrast-enhancing T2-hyperintense areas were high in both types of tumor compared to those in the contrast-enhancing areas and normal appearing white matter. We assume that the high f2 values in the noncontrast-enhancing T2-hyperintense areas are likely to reflect mostly perifocal vasogenic edema rather than tumor infiltration outside the enhancing lesion. Our result is consistent with the previous DWI study in which ADC could not be used to differentiate edema with infiltration of tumor cells from vasogenic edema in high-grade gliomas and PCNSLs [38].”

New references

36.	Law M, Cha S, Knopp EA, Johnson G, Arnett J, Litt AW. High-grade gliomas and solitary metastases: differentiation by using perfusion and proton spectroscopic MR imaging. Radiology. 2002;222(3):715-21. Epub 2002/02/28. doi: 10.1148/radiol.2223010558. PubMed PMID: 11867790.

37.	Neska-Matuszewska M, Bladowska J, Sasiadek M, Zimny A. Differentiation of glioblastoma multiforme, metastases and primary central nervous system lymphomas using multiparametric perfusion and diffusion MR imaging of a tumor core and a peritumoral zone-Searching for a practical approach. PLoS One. 2018;13(1):e0191341. Epub 2018/01/18. doi: 10.1371/journal.pone.0191341. PubMed PMID: 29342201; PubMed Central PMCID: PMCPMC5771619.

- Statistical Analysis
Page 9 – “The optimal cutoff point was determined by Youden's method.” – A literature reference should be provided to support the method.

**Response:** The reference for Yuden’s method has been provided here.

28.	Youden WJ. Index for rating diagnostic tests. Cancer. 1950;3(1):32-5. Epub 1950/01/01. doi: 10.1002/1097-0142(1950)3:1<32::aid-cncr2820030106>3.0.co;2-3. PubMed PMID: 15405679.

Page 10 – “To determine whether the combination of multiple parameters for both the GD model and the IVIM model increases the diagnostic performance, we first performed a stepwise analysis to select appropriate parameters, and then we performed a binomial logistic regression analysis to examine the combinations of the selected parameters.” – Does “appropriate” means with the best diagnostic performances? “Examine” in what way? This should be presented in a clearer way.

**Response:** We have provided more detailed explanations of the stepwise analysis in the paragraph as follows.

Materials and Methods (Page 10, Line 11-20)

“To determine whether the combination of multiple parameters for both the GD model and the IVIM model increases the diagnostic performance, we first performed a stepwise analysis to select the explanatory variables for a multiple regression model from a group of candidate variables by going through a series of automated steps. A forward-selection rule was applied in which the analysis started with no explanatory variables and then added variables, one by one, based on which variable was the most statistically significant, until there were no remaining statistically significant variables [30, 31]. We then performed a binomial logistic regression analysis to examine the AUCs of the combinations of the selected parameters. Two independent AUCs were compared using the method of Delong et al. [32].”

New references

30.	Efroymson MA. Multiple regression analysis. In: Ralston A, Wilf HS, editors. Mathematical methods for digital computers. New York: Wiley; 1960.

31.	Smith G. Step away from stepwise. J Big Data. 2018;5(32). doi: org/10.1186/s40537-018-0143-6.

• Results
- Comparisons of the parameters between the PCNSL and GB groups
Page 10 - 11 - “Figure 2 illustrates the results of our comparisons of the GD model-derived parameters between the PCNSLs and GBs in the gadolinium-enhancing lesions. The κ was significantly smaller in the PCNSL group (2.26 ± 1.00) than in the GB group (3.62 ± 2.01, p=0.0004). The θ was not significantly different between the groups. The f1 was significantly larger in the PCNSL group (0.542 ± 0.107) than in the GB group (0.348 ± 0.132, p<0.0001). The f2 was significantly smaller in the PCNSL group (0.372 ± 0.098) than in the GB group (0.508 ± 0.127, p<0.0001). The f3 was also significantly smaller in the PCNSL group (0.086 ± 0.043) than in the GB group (0.144 ± 0.062, p<0.0001). The detailed information for the parameters is summarized in Table 1. Figure 3 provides a PCNSL case that showed a ring-like enhancing mass lesion mimicking a GB. This lesion showed a low κ, a large f1, a small f2, and a small f3, suggesting PCNSL. Figure 4 demonstrates a GB case that showed a solid enhancing mass lesion. This lesion showed a small κ, a small f1, moderate f2 and large f3, which are consistent with GB. In the T2-hyperintense areas without contrast enhancement, no significant differences were found between the PCNSL and GB groups for any of the GD model derived parameters.” - This paragraph should be written in a more fluid way so the reader can better understand the ideas that the authors chose to highlight.

**Response:** This paragraph has been rewritten and reorganized in a following order: 1) the results for the gadolinium enhancing lesions, 2) the results for peritumoral T2-hyperintense areas without contrast enhancement, 3) the results for normal appearing white matter, 4) representative figures for PCNLS and GB.

Results (Page 11, Line 6 – Page 12, Line 9)

“The detailed information for the parameters in the gadolinium enhancing lesion, peritumoral T2-hyperintense areas without contrast enhancement, and normal appearing white matter is summarized in **Table 1**.

The results of our comparisons of the GD model-derived parameters between the PCNSLs and GBs in the gadolinium-enhancing lesions are shown in **Figure 2**. In the gadolinium-enhancing lesions, the κ was significantly smaller in the PCNSL group (2.26 ± 1.00) than in the GB group (3.62 ± 2.01, p=0.0004), the f1 was significantly larger in the PCNSL group (0.542 ± 0.107) than in the GB group (0.348 ± 0.132, p<0.0001), the f2 was significantly smaller in the PCNSL group (0.372 ± 0.098) than in the GB group (0.508 ± 0.127, p<0.0001), and the f3 was significantly smaller in the PCNSL group (0.086 ± 0.043) than in the GB group (0.144 ± 0.062, p<0.0001), while the θ was not significantly different between the groups.

In the peritumoral T2-hyperintense areas without contrast enhancement, no significant differences were found between the PCNSL and GB groups for any of the GD model derived parameters.

In the contralateral normal-appearing white matter, the f1 was significantly larger in the PCNSL group (0.642 ± 0.047) than in the GB group (0.593 ± 0.044, p<0.0001), the f2 was significantly smaller in the PCNSL group (0.316 ± 0.036) than in the GB group (0.354 ± 0.038, p<0.0001), and the f3 was significantly smaller in the PCNSL group (0.043 ± 0.026) than in the GB group (0.053 ± 0.019, p=0.0105).

**Figure 3** provides a PCNSL case that showed a ring-like enhancing mass lesion mimicking a GB. This lesion showed a low κ, a large f1, a small f2, and a small f3, suggesting PCNSL. **Figure 4** demonstrates a GB case that showed a solid enhancing mass lesion. This lesion showed a small κ, a small f1, moderate f2 and large f3, which are consistent with GB.”

Page 14 – The results T2-hyperintense areas without contrast enhancement were mentioned but no analysis was made to the NAWM results. If the results were obtained these should be used to support the information shown about the GD model in the manuscript.

**Response:** Thank you for pointing this out. We have added the statistical analyses on the NAWM. Unexpectedly, there were significant differences in the f1, f2, and f3 of NAWM between the PCNSLs and GB groups as follows. We have added the results and discussion on these differences as follows. I found some errors in the values for the NAWM in the GB group and have corrected them (the correction did not affect the statistical results).

Results (Page 11, Line 21- Page 12, Line 4)

“In the contralateral normal-appearing white matter, the f1 was significantly larger in the PCNSL group (0.642 ± 0.047) than in the GB group (0.593 ± 0.044, p<0.0001), the f2 was significantly smaller in the PCNSL group (0.316 ± 0.036) than in the GB group (0.354 ± 0.038, p<0.0001), and the f3 was significantly smaller in the PCNSL group (0.043 ± 0.026) than in the GB group (0.053 ± 0.019, p=0.0105).”

Discussion (Page 24, Line 4-9)

“In the normal-appearing white matter, the GB group showed larger f1, smaller f2, and larger f3 than the PCNSL group although these differences were small. This was unexpected, and the reasons for the differences remain unclear; however, since GBs frequently show extensive infiltration into the surrounding brain tissue, which is a fundamental feature of diffuse glioma, it is no wonder that the increased cell density and perfusion were observed in the normal-appearing white matter.”

Page 14 – In the "all data" excel sheet for f parameter you use percentage and here you show a fraction. Please make it uniform to clearly inform the reader.

**Response:** Thank you for pointing out. In the excel file named “all data”, the f-values have been expressed as a fraction.

Page 18 – “(…) although the difference was not significantly different from the values for D (p=0.5276).” – This should be rephrased.

**Response:** This has been rephrased as follows.

Results (Page 19, Line 11-12)

“..., although the AUC for this combination was not significantly different from that for D (p=0.5276).”

- Correlations of the model parameters
Page 18 – “The f1 had an almost perfect inverse correlation with D (all,
r = −0.976, p<0.0001; (…)” – According to figure 6, r = −0.9756, please confirm the information and change where necessary.

**Response:** We have rounded the r-values to four decimal places as follows.

Results (Page 19, Line 18 - Page 20, Line 3)

**“Figure 6** shows the correlations among the GD model-derived and IVIM model-derived parameters in all tumors. The f1 had an almost perfect inverse correlation with D (all, r = −0.9756, p<0.0001; PCNSL, r = −0.9558, p<0.0001; GB, r = −0.9699, p<0.0001). The f2 had a very strong positive correlation with D (all, r=0.8865, p<0.0001; PNCSL, r=0.9619, p<0.0001; GB, r=0.8273, p<0.0001). The f3 had a very strong positive correlation with the f (all, r=0.8654, p<0.0001; PNCSL, r=0.8317, p<0.0001; GB, r=0.8611, p<0.0001).”

Page 18 – Considering that the correlation between f2 and D parameters is 0.88657, and it is mentioned because it is high compared to the other correlations, the correlation between f1 and f2 should also be mentioned. The correlation between f1 and f2 is above 0.9155 and the meaning of this result can be of interest to be discussed in the appropriate section of this work.

**Response:** Thank you for the suggestion. Yes, there was a very strong negative correlation between f1 and f2. The following results and discussion regarding the relationship between f1 and f2 have been added. The negative correlation between f1 and f2 was likely due to the complementary relationship between these two parameters. Since the f3-values (PCNSL group, 0.086±0.043; GB group, 0.144±0.062) were smaller compared to the f1- (PCNSL group, 0.542±0.107; GB group, 0.348±0.132) and f2-values (PCNSL group, 0.372±0.098; GB group, 0.508±0.127), the increased f1 would result in the decreased f2, and vice versa.

Results (Page 20, Line 1-3)

“The f1 had an very strong negative correlation with the f2 (all, r = −0.9155, p<0.0001; PCNSL, r = −0.9150, p<0.0001; GB, r = −0.8874, p<0.0001).”

Discussion (Page 23, Line 5-7)

“The negative correlation between f1 and f2 was likely due to the complementary relationship between these two parameters. Since the f3-values were smaller compared to the f1- and f2-values, the increase in f1 would result in the decrease in f2, and vice versa.”

• Discussion
- The meaning of f3 and f parameters results and finding should be analysed in the discussion. Why f and f3 parameters are higher in the PCNSL when compared to the GB group of lesions?

**Response:** Thank you for your suggestion. Yes, this is an important point and we should have mentioned this. The larger f3 and f values in the GB group compared to those in the PCNSL group can be attributed to the difference in vascularity of these tumors. Pathologically, neovascularization is a key feature of GB while it is not prominent in PCNSL. Our results for f3 and f are consistent with those from previous studies using dynamic susceptibility contrast perfusion-weighted MR imaging and arterial spin labeling imaging. We have added the discussions on the meaning of f3 and f as follows.

“The GB group showed larger f3 and f compared to the PCNSL group. This may be attributed to the difference in vascularity of these tumors. Pathologically, neovascularization is a key feature of GB while it is not prominent in PCNSL [33, 34]. Our results are consistent with those from previous studies using dynamic susceptibility contrast perfusion-weighted imaging and arterial spin labeling imaging [9, 35].”

New references

33.	Hardee ME, Zagzag D. Mechanisms of glioma-associated neovascularization. Am J Pathol. 2012;181(4):1126-41. Epub 2012/08/04. doi: 10.1016/j.ajpath.2012.06.030. PubMed PMID: 22858156; PubMed Central PMCID: PMCPMC3463636.

34.	Cha S, Knopp EA, Johnson G, Wetzel SG, Litt AW, Zagzag D. Intracranial mass lesions: dynamic contrast-enhanced susceptibility-weighted echo-planar perfusion MR imaging. Radiology. 2002;223(1):11-29. Epub 2002/04/04. doi: 10.1148/radiol.2231010594. PubMed PMID: 11930044.

35.	Hartmann M, Heiland S, Harting I, Tronnier VM, Sommer C, Ludwig R, et al. Distinguishing of primary cerebral lymphoma from high-grade glioma with perfusion-weighted magnetic resonance imaging. Neurosci Lett. 2003;338(2):119-22. Epub 2003/02/05. doi: 10.1016/s0304-3940(02)01367-8. PubMed PMID: 12566167.

- Another important result, which should be discussed, is that with the GD model parameters (κ, f1, and f3) it was possible to obtain a higher AUC when compared to ADC’s AUC. Can the author provide a justification or further analysis?

**Response:** Thank you very much. As pointed out, the AUC of this combination (0.909) was higher than that of ADC (0.879). Although there was no statistically significant difference between them (p=0.2152), this tendency could be meaningful and important. Whether the combination of parameters of the GD model has an additive value should be evaluated in a larger population. We have added the following sentences in Results and Discussion.

Results (Page 19, Line 6-8)

“The AUC of this combination (0.909) was higher than that of ADC (0.879); however, there was no statistically significant difference between them (p=0.2152).”

Discussion (Page 22, Line 16-19)

“The AUC of this combination tended to be higher than that of ADC although there was no significant difference. Whether the combination of parameters of the GD model has an additive value should be evaluated in a larger population, since we did not observe statistical significance in all of our comparisons.”

Page 20 – “The reason for the slightly higher AUC observed with the ADC could be the effect of perfusion on ADC measurements.” - Can the effect of perfusion on ADC explain the higher AUC observed? If that is the case, in what way can this be explained?

**Response:** I am sorry that our discussion lacked explanations for this result. The ADC is a diffusion coefficient but is influenced by tissue perfusion (that's why this is called “apparent” diffusion coefficient). In hyperperfused tissues, ADC will be affected by perfusion and overestimated compared to true diffusion coefficient D, while the perfusion effect will be excluded in the calculation of D. In hypervascular tumors such as GBs, the ADC would be larger than D. On the other hand, in hypovascular tumors such as PCNSLs, this difference between ADC and D should be smaller. This means that the difference between ADC and D would be larger in GBs than in PCNSLs. Therefore, ADC could show higher diagnostic performance in the discrimination of these two tumors than D. This paragraph has been modified and added more explanations as follows.

Discussion (Page 22, Line 3-14)

“The reason for the slightly higher AUC observed with the ADC could be the effect of perfusion on ADC measurements. In hyperperfused tissues, ADC will be affected by the perfusion effect and overestimated compared to D; however, since both f1 and D are parameters without a perfusion effect in theory, an overestimation caused by perfusion should not be observed in these values. Therefoere, in hypervascular tumors such as GBs, the ADC should be larger than D. On the other hand, in hypovascular tumors such as PCNSLs, this difference between ADC and D should smaller. This means that the difference between ADC and D would be larger in GBs than in PCNSLs. Therefore, ADC could show higher diagnostic performance in the discrimination of these two tumors than D. In fact, the difference between the ADC and D values was greater in the GBs (0.100 × 10^−3^ mm^2^/sec) than in the PCNSLs (0.078 × 10^−3^ mm^2^/sec), which was most likely due to the higher perfusion effect on the ADC in GBs than in PCNSLs.”

Page 21 – “We found the correlations between the GD model-derived and IVIM-derived parameters.” – This sentence is vague or incomplete please consider revising it.

**Response:** This sentence has been revised to be more specific as follows.

Results (Page 22, Line 21-22)

“We found the correlations between the GD model-derived and IVIM-derived parameters, particularly between the f1 and D, the f2 and D, and the f3 and f.”

Page 21 – “The almost perfect correlation observed between the f1 and D may indicate that these two parameters contain virtually identical information.” – It would be important to mention that it is a negative correlation.

**Response:** The term “negative” has been added as requested.

Discussion (Page 22, Line 1)

“The almost perfect negative correlation observed between the f1 and D may indicate that these two parameters contain virtually identical information.”

Page 21 – “The positive correlation between f2 and D suggests the opposite, and the increased extracellular space like that taken up by microscopic necrosis might result in the higher f2.” – The “opposite” to what?

**Response:** This wording was not appropriate, and thus the term “opposite” has been deleted as follows.

Discussion (Page 23, Line 2-3)

“The positive correlation between f2 and D suggests that the increased extracellular space like that taken up by microscopic necrosis might result in the higher f2.”

Page 21 – “Although the GD-derived and IVIM-derived parameters provide similar information, the strength of the GD model-derived parameters is that the values are expressed as a fraction or percentage, which allows us to characterize
tumors from histological viewpoint.” - “f “ is an IVIM-derived parameter and it is also expressed in percentage or fraction.

**Response:** Yes, as you pointed out, the IVIM-f is expressed in percentage or fraction. But the IVIM method cannot express the fractions or percentage of intracellular and extracellular-extravascular spaces. Therefore, we think IVIM is not a perfect method to characterize tumors from histological viewpoint.

Page 21 – “The high f2 values in both types of tumor are likely to reflect mostly perifocal vasogenic edema rather than tumor infiltration outside the enhancing lesion” – “high f2 values” relative to what? The sentence would be more specific if it mentioned that it is referring to T2-hyperintense lesions. This idea would benefit from an addition of a literature reference.

**Response:** The f2 values in the noncontrast-enhancing T2-hyperintense areas were higher in both types of tumor compared to those in the contrast-enhancing areas and normal appearing white matter. The sentence has been revised as follows. This is a new finding and we could not find an appropriate reference related to this result. A new reference has been added.

Discussion (Page 23, Line 19 – Page 24, Line 4)

“The f2 values in the noncontrast-enhancing T2-hyperintense areas were higher in both types of tumor compared to those in the contrast-enhancing areas and normal appearing white matter. We assume that the high f2 values in the noncontrast-enhancing T2-hyperintense areas are likely to reflect mostly perifocal vasogenic edema rather than tumor infiltration outside the enhancing lesion. Our result is consistent with the previous DWI study in which ADC could not be used to differentiate edema with infiltration of tumor cells from vasogenic edema in high-grade gliomas and PCNSLs [38].

New refference

38.	Server A, Kulle B, Maehlen J, Josefsen R, Schellhorn T, Kumar T, et al. Quantitative apparent diffusion coefficients in the characterization of brain tumors and associated peritumoral edema. Acta Radiol. 2009;50(6):682-9. Epub 2009/05/19. doi: 10.1080/02841850902933123. PubMed PMID: 19449234.

Page 22 – Another limitation is the fact that only one person performed the ROI placement. The fact that the highest b-value in use was 1000s/mm2, should be better justified since in the brain higher b-values are usually used.

**Response:** The limitation for the ROI placement has been added. In a study of prostate cancers, Oshio et al. used the similar DWI parameters to ours and highest b-value of 1000s/mm^2^, and reported that the good fitting accuracy was observed in both cancer (R^2^=0.99226) and normal tissue (R^2^=0.99842). Their results indicated that DWI with the highest b-value of 1000 s/mm^2^ can be used for GD model analysis. We have modified the paragraph to justify the use of this b-value as follows.

Discussion (Page 24, Line 12-14)

“The only one person performed the ROI placements on a single slice, and not whole tumor volume was evaluated.”

Discussion (Page 24, Line 17- Page 25, Line 5)

“In addition, the selection of b-values has not yet been optimized. Prior studies of the GD model used the maximum b-values ranging from 1000 to 3000 sec/mm^2^ [18, 22-24]. In a study of prostate cancers, Oshio et al. used the similar DWI parameters to ours and the highest b-value of 1000 s/mm^2^, and reported that the good fitting accuracy was observed in both cancerous tissues (R^2^=0.99226) and normal tissues (R^2^=0.99842) [22]. Their result indicated that DWI with the highest b-value of 1000 s/mm^2^ can be used for GD model analyses; however, since it was reported that the non-monoexponential diffusion-related signal decay generally becomes more obvious over more extended b-value ranges, the maximum b value of 1000 sec/mm^2^ used in the present study might be lower than the optimal value. The optimal b-values and numbers should be elucidated in future studies.”

- Figures
Figure 1. It would be interesting to have the non-contrast-enhancing T2 image where the ROIs were also placed.

**Response:** Another case (new Figure 1 C, D) which well illustrates noncontrast-enhancing T2-hyperintense areas has been added.

Figure 5 The y axis title is missing

**Response:** Thank you very much! This has been corrected.

Figure 6 Units should be included, when appropriate.

**Response:** The units have been added for D and D*. The f1, f2, f3 and f are expressed as fractions and no units are necessary for them.

- Tables
Table 1 It would be easier to interpret the information shown in the table if p-values were also presented.

**Response:** The p-values have been added in Table 1 as suggested.

Table 2 In this table the reader is first exposed to the combination of parameters that were used to estimate diagnostic performances, additionally to the estimation of the diagnostic performances with the individual parameters. Why these specific combinations of parameters were considered? The justification should be clearly stated in the text.
In the legend “D, true diffusion coefficient; D*, ç; f, perfusion fraction” the meaning of D* is missing.

**Response:** The stepwise analysis selected the combination of κ, f1, and f3. This is already stated in the text as follows. The meaning of D* (pseudo-diffusion coefficient) has been corrected.

Results (Page 19, Line 1-4)

“In the combined-parameters analysis, the stepwise procedure selected κ, f1, and f3 for the GD model, and the D and f for the IVIM model. The combination of κ, f1, and f3 revealed excellent diagnostic performance with the AUC of 0.909, sensitivity of 84.2%, and specificity of 88.9%.”

- Supporting information
Units should be added to the “all data” table where appropriate.

**Response:** The units have been added in all data table.

----Statement:
The topic is interesting but there are some parts of the study which the description should be improved so the reader can better understand it.
The major strengths of the article are: the application of a new diffusion model to brain tumours, which as far as I know it has not been done yet; the inclusion of an analysis of combined parameters and the evaluation of its performance; the inclusion of healthy tissue results, but these results were not compared to the results obtain for tumours which is a weakness. Consequently, the relevant weaknesses of the work are: the need for a deeper analysis of the obtained results; there are some methods and procedures that should be better described and justified; the use of b=1000 s/mm2 as the highest b-value is unusual in a brain diffusion studies, and this should be further justified.

**Response:** We sincerely appreciate the reviewer’s positive comments. We believe that we have corrected all the weaknesses of the present study which the reviewer pointed out as seen above.

Note: “Have the authors made all data underlying the findings in their manuscript fully available?” My answer was “no” because I only had access to the summary statistics, and not to the data points behind the statistics.

**Response:** In the supplementary data named “all data”, all raw data of measurements are available. We forgot to include raw data for T2-hyperintense areas and NAWM in the previous file and have added them in the new file.

Reviewer #2: This study examined gamma distribution model of diffusion MRI and this model is useful to differentiate malignant lymphoma and glioblastoma.
This is well written paper and there are some minor points to revise.

In table 1, clarify the statistical significant differences between PCNSL and GB.

**Response:** We have added all p-values in Table 1 as suggested.

In figure 4, the k and f2 values at lateral peritumor area (lateral side) are high compared to contrast-enhanced tumor area.

**Response:** Yes, the peritumoral T2-hyperintense area without contrast enhancement showed a large κ and a large f2 compared to the contrast-enhancing area. These findings are consistent with the results of the present study (Table 1). We have added the descriptions about the peritumoral T2-hyperintense areas in Fig. 3 and 4. Thank you very much for your suggestion.

**Fig. 3.** The peritumoral T2-hyperintense area without contrast enhancement shows a large κ (8.18, **D**), a small θ (0.46×10^−6^ mm^2^/sec, **E**), a small f1 (0.139, **F**), a large f2 (0.772, **G**), and a small f3 (0.090, **H**).

**Fig. 4.** The peritumoral T2-hyperintense area without contrast enhancement shows a large κ (3.94, **D**), a small θ (0.75×10^−6^ mm^2^/sec, **E**), a small f1 (0.308, **F**), a large f2 (0.597, **G**), and a small f3 (0.095, **H**).

Discussion
Limitation. The authors evaluate the only one slice of tumor, not whole tumor volume. Add this point in the limitation.

**Response:** We agree with this point. This has been added in the limitation as follows.

Discussion (Page 24, Line 12-14)

“The only one person performed the ROI placements on a single slice, and not whole tumor volume was evaluated.”
