# Supplementary material for: Gamma distribution model of diffusion MRI for the differentiation of primary central nerve system lymphomas and glioblastomas
Source: PLoS One. 2020 Dec 14;15(12):e0243839. doi: 10.1371/journal.pone.0243839 (PMC7737570; doi:10.1371/journal.pone.0243839)
Supplement: Attachment — Submitted filename: PLOSONE_revise_2nd.docx [file pone.0243839.s003.docx]

Reviewer #1: The authors have addressed the topics indicated in the first revision and the manuscript has been significantly improved. Here, the authors can find some small details that still need to be considered.

• Results (Page 19, Line 11-12)
“..., although the AUC for this combination was not significantly different from that for D (p=0.5276).”
"This combination increased the diagnostic performance of f (p=0.0077), although the AUC for this combination was not significantly different from that for D (p=0.5276)."

- The language used in the phrases “(…) significantly different from that for (…)”through all the text should be rewritten in a clearer way in order to sound.

**Response:** First, we would like to thank you for your thorough reading and constructive criticism which greatly enhanced the quality of the paper. According to your comments, we have carefully revised the paper again. The sentences have been rewritten as follows.

“This combination increased the diagnostic performance of κ (p=0.0016), and f3 (p=0.0075), although it did not improve the performance of f1 (p=0.1950).” (Page 19, Line 4-6)

“This combination improved the diagnostic performance of f (p=0.0077), although it did not improve the performance of D (p=0.5276).” (Page 19, Line 9-10)

• Discussion (Page 23, Line 5-7)
“The negative correlation between f1 and f2 was likely due to the complementary relationship between these two parameters. Since the f3-values were smaller compared to the f1- and f2-values, the increase in f1 would result in the decrease in f2, and vice versa.”

- An explanation for this relation should be put forward taking into account the meaning of the parameters.

**Response:** The relationship between f1 and f2 has been explained considering the meaning of the parameters as follows.

“The negative correlation between f1 and f2 was likely due to the complementary relationship between these two parameters. In general, intravascular space (≒ f3) is smaller compared to intracellular (≒ f1) and extracellular extravascular space (≒ f2). In fact, the f3-values were much smaller than the f1- and f2-values in both PCNSLs and GBs in the present study. Therefore, the increase in f1 would result in the decrease in f2, and vice versa.” (Page 23, Line 4-9)


• “Page 21
“Although the GD-derived and IVIM-derived parameters provide similar information, the strength of the GD model-derived parameters is that the values are expressed as a fraction or percentage, which allows us to characterize tumours from histological viewpoint.” - “f “ is an IVIM-derived parameter and it is also expressed in percentage or fraction.
Response: Yes, as you pointed out, the IVIM-f is expressed in percentage or fraction. But the IVIM method cannot express the fractions or percentage of intracellular and extracellular-extravascular spaces. Therefore, we think IVIM is not a perfect method to characterize tumours from histological viewpoint.”

- In the way this sentence is presented, the reader may misunderstand the information that you are providing. You are stating that one strong point of the GD model parameters, when compared to IVIM parameters, is to be presented in percentage or fraction and that this is the reason why it allows the characterization of tumours. f is also expressed as fraction or percentage and it shows problems in this task. In the way that the sentence is constructed the reader may think that only GD parameters are expressed in fraction or percentage. Also, it is important to refer in what way can this characteristic contribute to the characterization of tumours' histology.

**Response:** Thank you very much for the comments. We have modified the sentences as follows according to your suggestion. We think that obtaining all fraction values (f1, f2, f3) would help to characterize tumors from histological viewpoint since f1, f2, and f3 should reflect cell density, interstitial space, and vascularity, respectively.

“Although the GD-derived and IVIM-derived parameters provide similar information, the strength of the GD model-derived parameters is that all fraction values (f1, f2, f3) are expressed as fractions or percentages, which allows us to well characterize tumors from histological viewpoint. The IVIM-derived f-value is also expressed in a percentage or fraction; however, the IVIM analysis is not able to provide the fraction values for intracellular and extracellular-extravascular spaces. In this sense, the IVIM method is not a perfect method for the histological characterization of tumors.”(Page 23, Line 9-15)

• Figure 1
- The name of the lesions should be included. Also, the lesions/ROIs in the images should be identified for example with numbers or letters, and that should be referenced and related in the legend of the figure.

**Response:** Thank you for your suggestions. The names of the lesions have been included in the legend. The ROI numbers (#1, contrast enhancing areal; #2 T2-hyperintense area; #3, normal appearing white matter) have been added in the Figures and have been related in the legends as follows.

**Fig. 1.** Regions-of-interest (ROIs). Figures 1 A and B show a GB with ring enhancement, and Figures C and D show a PCNSL with solid enhancement. The ROIs were placed on postcontrast T1-weighted images to include contrast enhancing lesions (**A**, **C**, area #1). The ROIs were also placed on the non-contrast-enhancing T2-hyperintense areas surrounding the contrast-enhancing area (area #2) and the contralateral normal-appearing white matter (**B**, **D**, area #3).

Reviewer #2: The paper is revised as reviewer's comments and it is acceptable in this version.
This is very useful information for brain tumor imaging.

**Response:** We appreciate the reviewer’s comments.
